# The Role of Heme and Reactive Oxygen Species in Proliferation and Survival of *Trypanosoma cruzi*

**DOI:** 10.1155/2011/174614

**Published:** 2011-10-09

**Authors:** Marcia Cristina Paes, Daniela Cosentino-Gomes, Cíntia Fernandes de Souza, Natália Pereira de Almeida Nogueira, José Roberto Meyer-Fernandes

**Affiliations:** ^1^Laboratório de Interação de Tripanossomatídeos e Vetores, Departamento de Bioquímica, IBRAG, UERJ, 20521-160 Rio de Janeiro, RJ, Brazil; ^2^Instituto Nacional de Ciência e Tecnologia-Entomologia Molecular (INCT-EM), 21941-521 Rio de Janeiro, RJ, Brazil; ^3^Laboratório de Bioquímica Celular, Instituto de Bioquímica Médica, Centro de Ciências da Saúde, UFRJ, 21941-521 Rio de Janeiro, RJ, Brazil; ^4^Instituto Nacional de Ciência e Tecnologia de Biologia Estrutural e Bioimagem, 21941-521 Rio de Janeiro, RJ, Brazil

## Abstract

*Trypanosoma cruzi*, the protozoan responsible for Chagas disease, has a complex life cycle comprehending two distinct hosts and a series of morphological and functional transformations. Hemoglobin degradation inside the insect vector releases high amounts of heme, and this molecule is known to exert a number of physiological functions. Moreover, the absence of its complete biosynthetic pathway in *T. cruzi* indicates heme as an essential molecule for this trypanosomatid survival. Within the hosts, *T. cruzi* has to cope with sudden environmental changes especially in the redox status and heme is able to increase the basal production of reactive oxygen species (ROS) which can be also produced as byproducts of the parasite aerobic metabolism. In this regard, ROS sensing is likely to be an important mechanism for the adaptation and interaction of these organisms with their hosts. In this paper we discuss the main features of heme and ROS susceptibility in *T. cruzi* biology.

## 1. *Trypanosoma cruzi* and Its Biological Cycle

*Trypanosoma cruzi* comprises a complex group of parasite populations circulating among humans, vectors, reservoirs, and wild and domestic animals [1]. This parasite is the causative agent of Chagas disease or American trypanosomiasis [2] and is transmitted through triatomine vectors, which are blood-sucking insects, when they feed on the vertebrate host.

After an insect feeds on the blood of an infected vertebrate, the development cycle of the parasite begins in the intestinal tract of triatomines. In the anterior midgut, most blood trypomastigotes transform into epimastigotes a few hours after ingestion. Some epimastigotes multiply by longitudinal binary fission, and in the insect rectum, a new differentiation occurs (metacyclogenesis process) in which epimastigotes are transformed into metacyclic trypomastigotes. These metacyclic trypomastigotes (highly infectious) are shed in feces and reach the bloodstream of a new vertebrate host after this host scratches an insect bite. The organisms penetrate the mucosa where there are many macrophages; after intense multiplication in the host cell in the form of amastigotes, they transform into trypomastigotes again, returning to the vertebrate circulation and completing the cycle [3]. These series of morphological and biochemical transformations in the life cycle may occur in response to external stimuli [4]. Recently, reactive oxygen species (ROS) and heme have been hypothesized to be important signaling molecules. In this way, protozoan parasites, which are specifically located in places where these molecules are constantly released, must evolve special mechanisms to take advantage of them. This paper will focus on the principal features of heme in *T. cruzi* biology and how different forms of these parasites are susceptible to ROS. 

## 2. Vectors of *Trypanosoma cruzi*

### 2.1. Biting Patterns and Importance of the Heme Molecule

Both the *Rhodnius prolixus* and *Triatoma infestans* species are very important in disease transmission. Differentiation into each of the five larval stages and the adult stage of these organisms is determined by their food. These blood-sucking insects ingest 6 to 12 times their original weight in blood. Usually, approximately 10 mM of heme bound to hemoglobin is obtained in a single ingestion [5]. 

Heme is a ubiquitous molecule usually associated with polypeptide chains through interactions between the iron atom and histidine or methionine residues. Heme catalyzes many oxidation processes in biological systems and is very important in cellular functions, as it is involved in oxygen transport (hemoglobin and myoglobin), in cellular respiration (cytochromes), in antioxidant defenses (peroxidases), in drug detoxification enzyme (P450), and cell signalling regulation (nitric oxide synthase) [6, 7]. Therefore, heme and hemeproteins are involved in basic functions such as oxygen sensitivity, cellular respiration, metabolism, growth, renewal, and cell differentiation, which are all essential for the survival of organisms. Moreover, heme is a toxic molecule due to its ability to generate reactive oxygen species and its amphiphilic features, to associate with lipid membranes, leading to altered membrane permeabilization and cell disruption [8–11].

### 2.2. Heme Uptake by *T. cruzi* Epimastigotes

The literature reports that there are some organisms that depend on essential hemeproteins but lack a heme biosynthetic pathway in part or in total; trypanosomatids are included in this group [12, 13]. In fact, biochemical and genomic studies have demonstrated the absence of key enzymes of heme biosynthesis in *T. cruzi* [14, 15]. In this regard, it is plausible that these parasites take up heme from the midgut of their vector.

In fact, during the development of *T. cruzi* epimastigotes in the digestive tract of insects, parasites utilize exogenous growth factors. Using ultrastructural studies, it has been noted that these factors are taken up by endocytosis via the flagellar pocket and the cytostome [16]. The cytostome, present in the anterior region of the cell near the flagellar pocket, is the preferred site of entry of bovine albumin and transferrin, and are reservosomes, the mature form of endosomes [17]. 

Interestingly, internalization of heme and hemoglobin proceeds through different routes and/or mechanisms. It has been shown through fluorescence microscopy using fluorescent heme analogues that entry is not modified by lowering the temperature, by preincubation with unlabeled hemoglobin or by reduction of ATP production. On the other hand, the transport of heme is compromised by the addition of cyclosporine, an inhibitor of ATP Binding Cassette- (ABC-) type transporters. This is the first evidence that heme uptake involves the activity of a P-glycoprotein (Pgp) homologue, an ABC transporter [18]. It has also been inferred that other insect trypanosomatids including *Crithidia deanei*, *Crithidia oncopelti,* and *Blastocrithidia culicis *obtain haem from their bacterial endosymbionts [19].

### 2.3. The Intracellular Trafficking of Heme in *T. cruzi* Epimastigotes

Although fluorescent analogue of heme is internalized faster than hemoglobin in *T. cruzi*, suggesting the existence of two different pathways used to target molecules, the intracellular traffic of heme is the same as other various molecules, including several proteins [18, 20]. Heme internalization starts at the cytostome, involving vesicles that travel along the cell body (early endosomes), and ends at the reservosomes, which have been described as a site for protein and lipid accumulation [18].

### 2.4. Heme as a Signaling Molecule in * T. cruzi * Proliferation

Malaquias and Oliveira [21] showed that, when exposed to mitogenic factors present in fetal serum, *T. cruzi* cells are stimulated by phosphoinositide-specific phospholipase C (PI-PLC), leading to the accumulation of phosphatidylinositol 3-phosphate (IP3) and diacylglycerol (DAG) and increasing their proliferation. Recent studies have shown the importance of second messengers in differentiation of trypanosomatids, including *T. brucei*-induced cAMP (adenosine 3′-5′ cyclic monophosphate) [22]. Several serine/threonine kinases, including a cyclin-dependent kinases [23] and a cAMP-dependent protein kinase (PKA) [24], phosphatidylinositol 3-kinase [25], a calcium-dependent protein kinase (PKC) [26], and a kinase dependent on calcium/calmodulin [27], have been identified in *T. cruzi* epimastigotes through biochemical studies, and in some cases, through molecular studies as well, such as PKA [28, 29]. The identification of these kinases groups has been corroborated by Parsons et al. [30]. Also in *T. cruzi*, before the differentiation of epimastigotes into metacyclic trypomastigotes, cAMP levels increase three- to fourfold inducing the differentiation of *T. cruzi* epimastigotes to metacyclic trypomastigotes [31].

It has been shown that heme, but not hemoglobin or its peptides, stimulates *T. cruzi* proliferation *in vitro *in a dose-dependent manner. Different strains were tested (Y and Dm28c), and both increased in the same manner. Further, a wide heme concentration range was employed, and even at higher concentrations, cells proliferated following the heme addition [18]. The authors hypothesized that heme could drive *T. cruzi *proliferation through a kinase cascade. 

Heme-induced growth of epimastigotes is not affected by inhibitors of cGMP-dependent protein kinase (PKG), PKC, PKA, PI3K, or cyclin-dependent kinase. Moreover addition of KN 93 and Myr-AIP (inhibitors of calmodulin kinase) to a culture of these cells reduces the expected growth, indicating the involvement of calmodulin kinase in heme-mediated cell signaling [32]. Furthermore, the authors showed that heme-induced *T. cruzi* growth is associated with CaMKII [31], demonstrating a signaling role for the heme molecule in the biological cycle of *Trypanosoma cruzi*. Recently, heme was shown to modulate a (Na^+^ + K^+^) ATPase, via heme receptor-mediated stimulation of the PI-PLC/PKC signaling pathway in *Leishmania amazonensis *[33]. On the other hand, in *Trypanosoma brucei brucei *this biomolecule may be involved in nutritional control; it was able to inhibit activity of ectonucleoside triphosphate diphosphohydrolases (E-NTPDases), an enzyme that is involved in the generation of free adenosine outside of the cell, together with ecto-5′-nucleotidase [34]. 

### 2.5. Redox Metabolism of *T. cruzi*

As a protozoan parasite of vertebrate and invertebrate hosts, *T. cruzi* is susceptible to a number of oxidative killing mechanisms, including reactive oxygen species (ROS). ROS can be produced during the degradation of hemoglobin in the midgut of insect vector as a consequence of the release of high amounts of heme or as a byproduct of *T. cruzi* aerobic metabolism [5, 35]. During respiration, molecular oxygen can undergo partial reduction, giving rise to relatively stable species, by accepting one, two, or three electrons, with the formation of superoxide anions O_2_^•−^, hydrogen peroxide (H_2_O_2_), and hydroxyl radicals (^•^OH), respectively [36]. High rates of O_2_^•−^ can also be produced by the NADPH oxidase complex, which becomes active immediately after phagocytosis by macrophages. Superoxide radicals can also generate the formation of H_2_O_2_ by spontaneous or SOD-catalyzed dismutation [37]. 

The mitochondria are the main source of ROS generation in most eukaryotic cells. Mitochondrial ROS are recognized as the key element in cell signaling processes and in a variety of degenerative mechanisms [38]. The Trypanosomatidae family is characterized by a single long mitochondrion with a dilated region known as the kinetoplast, in which mitochondrial DNA (kDNA) is observed [39]. Despite these peculiar characteristics, mitochondria of trypanosomatids are able to generate and sustain a membrane potential comparable to mammalian mitochondria [40]. 

Like other trypanosomatids, *T. cruzi* has an intricate antioxidant defense system that varies with their life stages and is distinct from its mammal host and insect vector in its complexity. In contrast to their hosts, trypanosomatids lack GSH/glutathione reductase (GR) and thioredoxin/thioredoxin reductase systems. Their redox metabolism depends on a particular dithiol called trypanothione and its corresponding reductase, trypanothione reductase (TryR). Moreover, *T. cruzi *lacks catalase and glutathione peroxidase (GPx), two major eukaryotic enzymes employed in the detoxification of peroxides. In spite of the absence of these two enzymes, *T. cruzi* possesses two peroxiredoxins, an ascorbate-dependent hemoperoxidase, several distinct peroxidases, of which at least two share sequence homology with GPx, and four iron-containing superoxide dismutases (SOD). For a complete review of redox metabolism in *T. cruzi*, see [41]. A controlled balance between extracellular ROS production and the ability of the cell to deal with these oxidants may predict the success of certain life stages in colonization and survival within the host.

## 3. Differential Susceptibility of **T. cruzi ** Life Stages to ROS

*T. cruzi* responds differently to oxidative stress depending on its life stage. Analysis of 10 strains from *T. cruzi* demonstrated a significant increase in trypanothione synthetase (TryS) and in cytosolic and mitochondrial tryparedoxin peroxidase isoforms, during differentiation from the noninfective epimastigote to the infective metacyclic trypomastigote form. Moreover, these elevations in antioxidant enzymes were shown to be more pronounced in the virulent strains than in attenuated ones [42]. At the same time, ascorbate peroxidase and TryR remained unchanged during the different life stages of the parasite [42].

These differences in sensitivity correlated with the genetic diversity between the lineages *T. cruzi*. A comparative study between two different strains of *T. cruzi*, the Tulahuen strain (*T. cruzi* VI [43], previously classified as *T. cruzi* I) and the Y strain (*T. cruzi* II), showed significant differences in the resistance to H_2_O_2_ treatment. Epimastigote forms of Tulahuen strain were shown to be more resistant to H_2_O_2_ than Y strain, presenting higher activity of glucose-6-phosphate dehydrogenase (G6PDH), an enzyme implicated in the supply of NADPH, due to proper function of the trypanothione-dependent system and an increase in the cytosolic tryparedoxin peroxidase (TcCPx) content [35, 44]. Interestingly, Tulahuen strain was shown to be more susceptible to benznidazole, a well known prooxidant trypanocidal drug, than Y strain [45, 46]. In agreement with these findings, the *T. cruzi* I strains Col1.7G2 and Silvio X-10 cl1 displayed more resistance to H_2_O_2_ treatment than the *T. cruzi* II strains JG and Esmeraldo cl3. Nevertheless, in contrast to the phenotypes observed with the Y and Tulahuen strains, these differences could not be attributed to differences in the redox potential of the strains analyzed [47]. In this case, the differential sensitivity to oxidative stress was suggested to be due to changes in the activity of MSH2, a central component of the DNA mutation and mismatch repair (MMR) machinery [47]. The MMR has a key function in recognizing and repairing base mismatches and frame shift mismatches that escape DNA polymerase proofreading during DNA replication [48]. 

With respect to life stage, *T. cruzi* epimastigotes seem to be more susceptible to the generation of H_2_O_2_ resulting from xanthine oxidase activity in the serum of chagasic patients lacking a complement system. The treatment of epimastigotes with this serum resulted in an inhibition of cell growth *in vitro*, a decrease in SOD activity, and an increase in membrane lipid peroxidation. Interestingly, the same results were not observed when parasites were treated with serum from healthy individuals [49]. Why epimastigotes have higher susceptibility to human serum than infective forms is unclear, but the exposure of epimastigotes to fresh human serum over a short period of time led to a decrease in cell respiration, loss of mitochondrial membrane potential, increased O_2_^•−^ production, and release of cytochrome *c*, a process characteristic of programmed cell death [50]. Moreover, it seems that the mitochondrion has a fundamental role in epimastigote-dependent complement activation; an accumulation of high amounts of Ca^2+^ inside the mitochondrial matrix was observed, causing partial dissipation of the inner membrane potential and O_2_^•−^ production [51]. In addition to causing an endogenous increase of O_2_^•−^ during contact of epimastigotes with human serum, this noninfective stage also triggers O_2_^•−^ formation by macrophages, with high, almost lethal, toxicity to the cell because of the formation of peroxynitrite [52–54]. On the other hand, infective trypomastigotes may be less exposed to peroxynitrite, as these forms may not stimulate the respiratory burst efficiently [54]. Nevertheless, internalization of *T. cruzi* trypomastigotes by macrophages may activate NADPH oxidase, which is involved in O_2_^•−^ production and peroxynitrite formation. These forms also showed a higher sensitivity to peroxynitrite than to H_2_O_2_ [37]. Conversely, Tanaka et al. [55] demonstrated that H_2_O_2_ is the main oxygen metabolite responsible for killing *T. cruzi* inside macrophages, and *T. cruzi* trypomastigotes were shown to be more resistant to killing by H_2_O_2_ than were the epimastigotes. The LD_50_ at which epimastigotes were killed was 6.0 nmol/min/ml of H_2_O_2_, while the LD_50_ for trypomastigotes was 8.7 nmol/min/ml of H_2_O_2_ *in vitro* [55]. Exposure of metacyclic trypomastigotes to 70 *μ*M H_2_O_2_ for 6 h caused an increase of 46-fold in G6PDH specific activity, while G6PDH activity from epimastigote forms presented a time-dependent decrease at the same conditions [56]. It seems that trypomastigotes are more resistant to killing by activated primary macrophages or by increased oxygen radicals than epimastigotes [55]. Nevertheless, overexpression of epimastigote TcCPX increased parasite virulence and resistance to macrophage killing [57]. In trypomastigotes, the increased expression of antioxidant enzymes may also be involved to the persistence of these forms in the serum [41, 58, 59] or inside macrophages during phagocytosis. In agreement with this thought, trypomastigotes with TcCPx overexpressed, caused an increase in parasitemia and tissue inflammation during mouse infections [37]. More severe infections were also observed within metastatic forms of *Leishmania*; this was suggested to be due to the functional activity of peroxiredoxin [60]. Therefore *T. cruzi* antioxidant defense could be considered an important virulence factor [37, 57]. 

Although *T. cruzi* epimastigotes are shown to be more sensitive to ROS, these cells can tolerate various levels of oxidants. Pretreatment of *T. cruzi* epimastigotes with low H_2_O_2_ concentrations (15–20 *μ*M) allowed an increase in cell proliferation of parasites, accompanied by a transient adaptation response to higher H_2_O_2_ concentrations [35]. Moreover, transient oxidative stress can also induce *T. cruzi* epimastigotes growth by heme stimulation via a mechanism mediated by a CaM Kinase II-like pathway [61]. This adaptation mechanism could be related to increasing expression levels of arginine kinase, an enzyme involved in the interconversion between phosphoarginine, a molecule with high energetic potential like creatine, and ATP. The process was suggested to be independent of redox content, indicating the participation of an unknown stress response mechanism [62].

## 4. Redox Metabolism and Drug Resistance

The drugs currently used against Chagas disease are nifurtimox and benznidazole, two nitro chemotherapeutic agents described to have trypanocidal effects and an ability to generate ROS. ROS generation may occur through the reduction of the nitro group by the action of nitro anion radical or hydronitroxide radical which then may react with molecular oxygen generating O_2_^•−^. [63, 64]. Susceptibility of *T. cruzi* to nifurtimox and benznidazole has been described as correlated with the levels of free and conjugated glutathione [63, 65]. Treatment of *T. cruzi* cultures with nifurtimox or benznidazole resulted in a loss of reduced thiol compounds (GSH, trypanothione and glutathionyl spermidine), which was suggested to be probably due to the conjugation of these compounds with reduced metabolites of the nitro drugs rather than an oxidation effect on thiol consumption [65, 66]. Besides, it was also observed that redox-cycling activity of nifurtimox was only acquired at high concentrations doses (>400 *μ*M), two orders of magnitude higher than that required for antiproliferative activity [66]. Moreover, nifurtimox has been reported to act as an inhibitor of *T. cruzi* trypanothione reductase, an enzyme responsible for the maintenance of reduced state of the intracellular thiols [67, 68]. The total amount of free or conjugated glutathione may vary greatly either between different strains of *T. cruzi* or between the different life stages of a unique strain, with the following differentiation sequence: epimastigote > trypomastigote > amastigote [64]. These differences in thiol contents could explain the diversity in resistance of *T. cruzi* stages to treatment with these drugs and in its sensibility to ROS. The resistance of *T. cruzi* to trypanocidal drugs can also be associated with higher expression of enzymatic antioxidants. *In vitro*-induced *T. cruzi* resistant to benznidazole presented an increase in the expression of the cytosolic and mitochondrial tryparedoxin peroxidase isoforms and in FeSOD isoforms, compared with the correspondent sensitive lineage. [69–71]. Interestingly, these observations were not involved in naturally resistant strains [69–71]. In *T. brucei*, null mutants for SODB1 (cytosolic, glycosomal) exhibited 3-fold increased susceptibility to nifurtimox than wild-type cells [72]. It seems that nifurtimox and benznidazole divert thiol compounds from their ability to act as free radical scavengers. Therefore the protection against the toxic effects of ROS might rely on enzymatic antioxidant activities. Antioxidant defenses and drug susceptibility of most studied *T. cruzi* strains are summarized in Table 1. 

With respect to the search for alternative drugs for Chagas disease, the activity of natural compounds such as naphthoquinones, natural products of several families of higher plants, has been extensively investigated [73]. Like nifurtimox and benznidazole, the cytotoxicity of naphthoquinones has been implicated in redox cycling and ROS generation [74]. Recently, three derivative compounds of C-allyl lawsone (2-hydroxy-3-allyl-1,4-naphthoquinone) were shown to be effective against intracellular amastigotes, decreasing the percentage of infection in murine macrophages, with low toxicity to host cells. Indeed, it seems that this compound is involved in mitochondrial damage, accompanied by an increase in H_2_O_2_ generation. Epimastigotes were shown to be more resistant than trypomastigotes to treatment with these compounds, despite having a more sensitive mitochondrion and higher accumulation of H_2_O_2_ within the cells [75]. Another type of naphthoquinone, an *α*-lapachone derivative, was shown to have a trypanocidal effect. This compound was tested against two strains of *T. cruzi*, the Y strain (*T. cruzi *II) and the Colombian strain (*T. cruzi* I), which presented higher resistance to the treatment than Y. In line with these findings, the Y strain is partially resistant and the Colombian strain is highly resistant to the chemotherapeutic agents currently in use. In contrast with the other naphthoquinones, this *α*-lapachone derivative does not have the capacity to generate free radicals, instead, it might function in the inhibition of proteinases [76]. Although sensibility to ROS may be related to the different *T. cruzi* groups, *in vitro* analysis of prooxidant drugs susceptibility between *T. cruzi* I and *T. cruzi* II strains showed no significant differences [45, 46]. However, correlation between the susceptibility to benznidazole in distinct genetic groups of *T. cruzi* has been described [77]. The subject is still controversial, and in this case, geographic distribution and phylogenetic distances of parasites must be considered [45]. 

## 5. Conclusions

*T. cruzi* epimastigotes present divergent behavior after exposure to oxidative stress. It seems that these cells can deal with external addition of H_2_O_2_, on the other hand, the generation of ROS from mammalian sites like serum and the immune system represents a challenge to these cells. For example, membrane-bound phosphatases from *T. cruzi* are more resistant to the addition of sublethal doses of hydrogen peroxide than *Trypanosoma rangeli* phosphatase [78]. Interestingly, the concentrations of trypanothione vary between 1.52–2.1 mM in epimastigotes, 0.5 mM in trypomastigotes, and 0.12 mM in amastigotes [41]. At the same time, there is an increase in the expression of antioxidant enzymes during the differentiation of *T. cruzi* from a noninfective form to the infective form, trypomastigotes [41, 58]. In contrast, stress-induced oxidant resistance in *Leishmania chagasi* is not accompanied by an increase in ROS scavengers, but instead is suggested to be associated with heat shock proteins like HSP70 [79, 80]. The mechanism by which *T. cruzi* escape from the oxidative burst of mammalian macrophages is still unknown; nevertheless, exposure of phosphatidylserine on the surface of trypomastigotes surface induces a deactivating effect on macrophages. This profile is also seen in apoptotic cells as a means to avoid anti-inflammatory responses [67]. 

There is a growing interest in understanding how ROS could be involved in the signaling process that permits parasites to colonize such different microenvironments. In this way, especially for *T. cruzi*, the extreme heterogeneity of this population and its susceptibility to oxygen metabolites must be carefully noted. These observations, in addition to their epidemiological significance, could permit the development of more effective drugs for the treatment of Chagas disease.

## Figures and Tables

**Table 1:** Antioxidant defenses and drug susceptibility of *T. cruzi* strains.

Strain	DTU*	Redox state	Drug susceptibility	Reference
Y	II	Low concentrations of antioxidant enzymes; lower activity of G6PDH	Resistant	[42, 44]
Tulahuen	VI	High concentrations of antioxidant enzymes; higher activity of G6PDH; lower Thiol contents	Susceptible	[42, 44, 65]
Colombiana	I	High concentrations of antioxidant enzymes; no alteration of TcCPX and TcMPX and FeSOD isoforms	Resistant (natural)	[42, 69–71]
Cl Brener	VI	No alteration of TcCPX and TcMPX and FeSOD isoforms	Susceptible (*in vitro*)	[69–71]

*Discrete typing units (DTUs), according to Zingales et al., 2009 [43].
